# WebRASP: a server for computing energy scores to assess the accuracy and stability of RNA 3D structures

**DOI:** 10.1093/bioinformatics/btt441

**Published:** 2013-08-07

**Authors:** Tomas Norambuena, Jorge F. Cares, Emidio Capriotti, Francisco Melo

**Affiliations:** ^1^Facultad de Ciencias Biologicas, Departamento de Genetica Molecular y Microbiologia, Pontificia Universidad Catolica de Chile, Alameda 340, ^2^Molecular Bioinformatics Laboratory, Millennium Institute on Immunology and Immunotherapy, Santiago, Chile and ^3^Division of Informatics, Department of Pathology, University of Alabama at Birmingham, 619 19^th^ st. south, Birmingham, AL 35249, USA

## Abstract

**Summary:** The understanding of the biological role of RNA molecules has changed. Although it is widely accepted that RNAs play important regulatory roles without necessarily coding for proteins, the functions of many of these non-coding RNAs are unknown. Thus, determining or modeling the 3D structure of RNA molecules as well as assessing their accuracy and stability has become of great importance for characterizing their functional activity. Here, we introduce a new web application, WebRASP, that uses knowledge-based potentials for scoring RNA structures based on distance-dependent pairwise atomic interactions. This web server allows the users to upload a structure in PDB format, select several options to visualize the structure and calculate the energy profile. The server contains online help, tutorials and links to other related resources. We believe this server will be a useful tool for predicting and assessing the quality of RNA 3D structures.

**Availability and implementation:** The web server is available at http://melolab.org/webrasp. It has been tested on the most popular web browsers and requires Java plugin for Jmol visualization.

**Contact:** fmelo@bio.puc.cl

## 1 INTRODUCTION

In the recent years, there has been a shift in the way RNA molecule is conceived. It is now known that thousands of RNA species are transcribed in a genome serving as regulatory elements without coding for proteins. There are numerous such non-coding RNAs in the cell, many with unknown functions (Aalto and Pasquinelli, 2012; Guil and Esteller, 2012; Lee, 2012). Experimental determination of RNA 3D structures has hence become an essential tool for characterizing their functional activity. As a consequence, the number of RNA molecules deposited in the structural databases is increasing steeply (Capriotti and Marti-Renom, 2008). However, there is still a large gap between the number of structures solved at the atomic level and the available RNA sequences (Capriotti and Marti-Renom, 2008). To help reducing this gap, several approaches and tools for predicting RNA 3D structures have been recently developed (Das and Baker, 2007; Flores and Altman, 2010; Frellsen *et al.*, 2009; Jonikas *et al.*, 2009; Parisien and Major, 2008; Popenda *et al.*, 2012; Rother *et al.*, 2011; Sharma *et al.*, 2008). These developments necessitate RNA structure accuracy assessment methods and tools (Bernauer *et al.*, 2011; Capriotti *et al.*, 2011; Jonikas *et al.*, 2009). In this work, we introduce the web server WebRASP to assess RNA 3D structures. The assessment relies on a knowledge-based potential for scoring RNA structures based on distance-dependent pairwise atomic interactions (Capriotti *et al.*, 2011). We believe our tool efficiently distinguishes between accurate and inaccurate RNA 3D structures (Capriotti *et al.*, 2011).

## 2 IMPLEMENTATION

The core of the server relies on the Ribonucleic Acids Statistical Potential (RASP), recently developed and validated by comparison with experimental data (Capriotti *et al.*, 2011). RASP is a knowledge-based potential for scoring RNA structures based on distance-dependent pairwise atomic interactions. A standalone version of RASP, implemented in the C++ programming language, can be downloaded from the web server.

There are four different RASP potentials: coarse-grain, backbone, backbone-ribose and full-atom. The *coarse-grain potential* takes into account only the interactions between C3’ carbons (i.e. four atom types, one for each nucleotide). *Backbone potential* describes the energy scores considering the interactions between atoms O3’, C3’, C4’, C5’, O5’, P, OP1, OP2 and OP3 (36 atom types, 9 for each nucleotide). *Backbone and ribose potential* describe the energy score based on the interaction between atoms of the backbone (as described above) and the rest of the ribose atoms, namely, C1’, C2’ and O4’ (48 atom types, 12 for each nucleotide). Finally, *full atom potential* contains a definition of energy score for pairwise interactions of all non-hydrogen atoms in the four canonical nucleotides.

**Fig. 1.**
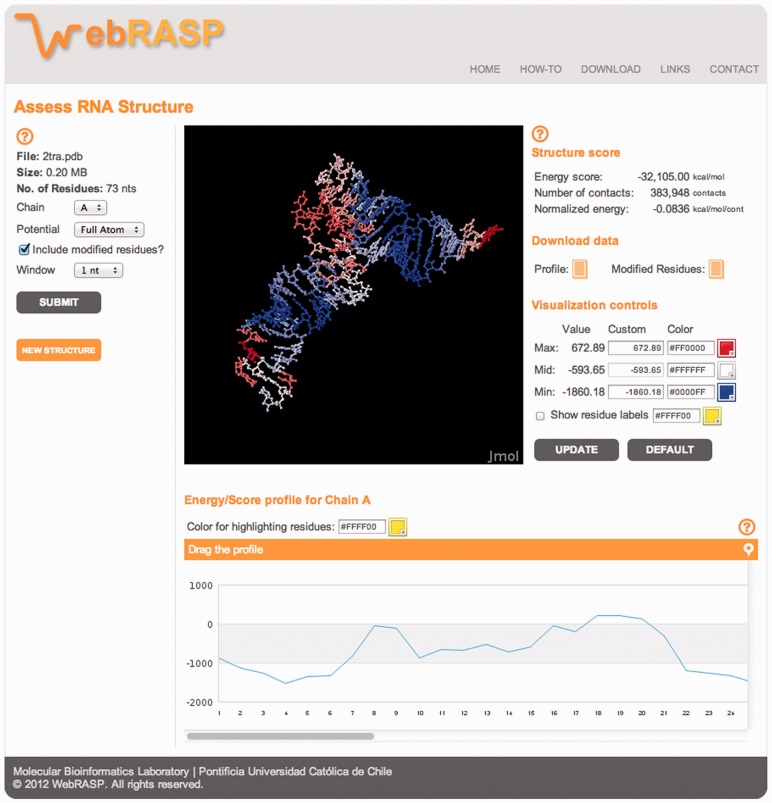
Screen snapshot of the assessment result for an RNA 3D structure

The energy score and profile of RNA molecules are computed in two sequential steps. First, a PDB file containing the atomic coordinates of the RNA structure is uploaded. Then, some general informative data about the uploaded structure is displayed, such as the name of the file, its size and the number of residues it contains. At this point, users can select the specific chain of the structure to be profiled, the potential to be used and the window size to average the energy scores. Additionally, in those RNA structure chains that contain non-canonical nucleotides, an option for including those non-standard nucleotides in the total energy score and energy profile calculation is available (this feature is only available on the web server, not in the standalone version). The atom type definition in this case is adopted based on the closest standard nucleotide. It is important to note that energy calculations for non-standard or modified residues are only approximate and could miss some undefined atom types (Fig. 1, left).

The results can be viewed graphically with the option of interactive modification. The information displayed is as follows: total energy score, number of contacts (the total number of pairwise atomic contacts occurring in the structure and used to calculate the total energy score) and normalized energy (the energy score value per atomic contact). Users can download associated data, namely, *Profile*, which is a text file containing the values of the energy score per residue; and, according to the options selected, *Modified residues*, which is a text file containing the non-canonical residues found in the structure. On the other hand, the *Visualization controls* provide the color scheme for controlling the visualization of the structure displayed in the Jmol applet. There are three columns: value, custom and color. Column *Value* lists the calculated maximum and minimum score values of the energy profile; the *Mid* value is the average between both values. Column *Custom* allows the users to modify the color scale by assigning different values to the maximum and minimum scores. Please note that the ‘Mid’ value cannot be customized because it is automatically adjusted. Column *Color* allows the users to select the colors for the gradient. The default color gradient goes from red (high-energy or unstable residues) to blue (low-energy or stable residues), going through white (residues of intermediate stability or accuracy). The option *Show residue labels* shows the name of the residues in the structure as text labels (users can also select the color for the labels). For any change of the visualization controls to take effect in the Jmol applet display window, users must use the *UPDATE* button (Fig. 1, right).

At the bottom, the *Energy/score profile* is displayed. According to the chain ID selected, this is the energy score profile per residue in that chain. The ordinate represents the energy score, whereas the x-axis represents the residue number. Rolling the mouse over the energy profile highlights the corresponding residue on the Jmol structure visualization applet. Users can also select the color shown for this highlight. The profile can be dragged off and horizontally scrolled to facilitate the interaction and visualization of the structure and the energy profile for long RNA molecules (Fig. 1, bottom). The server contains help links, a tutorial and a couple of pre-loaded examples.

*Funding*: This research was funded by grants from FONDECYT (No. 3210007 and 1110400) and ICM (Iniciativa Científica Milenio, Chile; No. P09-016-F). E.C. is supported by start-up funds from the Department of Pathology at the University of Alabama, Birmingham.

*Conflict of Interest*: none declared.

## References

[btt441-B1] Aalto AP, Pasquinelli AE (2012). Small non-coding RNAs mount a silent revolution in gene expression. Curr. Opin. Cell Biol..

[btt441-B2] Bernauer J (2011). Fully differentiable coarse-grained and all-atom knowledge-based potentials for RNA structure evaluation. RNA.

[btt441-B3] Capriotti E, Marti-Renom MA (2008). Computational RNA structure prediction. Curr. Bioinform..

[btt441-B4] Capriotti E (2011). All-atom knowledge-based potential for RNA structure prediction and assessment. Bioinformatics.

[btt441-B5] Das R, Baker D (2007). Automated de novo prediction of native-like RNA tertiary structures. Proc. Natl Acad. Sci. USA.

[btt441-B6] Flores SC, Altman RB (2010). Turning limited experimental information into 3D models of RNA. RNA.

[btt441-B7] Frellsen J (2009). A probabilistic model of RNA conformational space. PLoS Comput. Biol..

[btt441-B8] Guil S, Esteller M (2012). Cis-acting noncoding RNAs: friends and foes. Nat. Struct. Mol. Biol..

[btt441-B9] Jonikas MA (2009). Coarse-grained modeling of large RNA molecules with knowledge-based potentials and structural filters. RNA.

[btt441-B10] Lee JT (2012). Epigenetic regulation by long noncoding RNAs. Science.

[btt441-B11] Parisien M, Major F (2008). The MC-Fold and MC-Sym pipeline infers RNA structure from sequence data. Nature.

[btt441-B12] Popenda M (2012). Automated 3D structure composition for large RNAs. Nucleic Acids Res..

[btt441-B13] Rother M (2011). ModeRNA server: an online tool for modeling RNA 3D structures. Bioinformatics.

[btt441-B14] Sharma S (2008). iFoldRNA: three-dimensional RNA structure prediction and folding. Bioinformatics.

